# Computational methods to explore chromatin state dynamics

**DOI:** 10.1093/bib/bbac439

**Published:** 2022-10-07

**Authors:** Elias Orouji, Ayush T Raman

**Affiliations:** Epigenomics Lab, Princess Margaret Cancer Centre, University Health Network, Toronto, ON, Canada; Department of Genomic Medicine, University of Texas MD Anderson Cancer Center, Houston, TX, USA; Broad Institute of MIT and Harvard, Cambridge, Massachusetts, USA; Department of Biostatistics, Harvard T.H. Chan School of Public Health, Cambridge, Massachusetts, USA

**Keywords:** chromatin, histone modification, epigenomics, chromatin state

## Abstract

The human genome is marked by several singular and combinatorial histone modifications that shape the different states of chromatin and its three-dimensional organization. Genome-wide mapping of these marks as well as histone variants and open chromatin regions is commonly carried out via profiling DNA–protein binding or via chromatin accessibility methods. After the generation of epigenomic datasets in a cell type, statistical models can be used to annotate the noncoding regions of DNA and infer the combinatorial histone marks or chromatin states (CS). These methods involve partitioning the genome and labeling individual segments based on their CS patterns. Chromatin labels enable the systematic discovery of genomic function and activity and can label the gene body, promoters or enhancers without using other genomic maps. CSs are dynamic and change under different cell conditions, such as in normal, preneoplastic or tumor cells. This review aims to explore the available computational tools that have been developed to capture CS alterations under two or more cellular conditions.

## Graphical Abstract

**Graphical Abstract:**
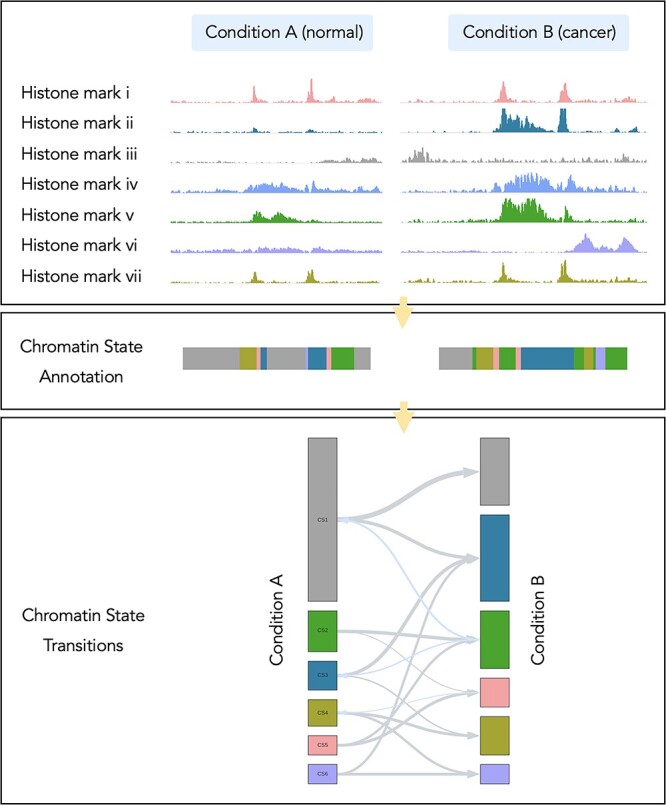

## Chromatin states

Chromatin is composed of DNA that is wrapped around proteins and condensed to fit into the cell nucleus. The fundamental unit of chromatin is the nucleosome, which is made up of eight histone proteins around which 147 bp of DNA is coiled. Histone tails can be altered with several different modifications, such as methylation and acetylation [1]. This could impact the tight or loose packaging of DNA. Each of these modifications at various positions on the histone tail is an individual histone mark. The human genome is marked by a series of these histone alterations. Any given region of the genome could be marked with no to multiple histone marks [2]. Some histone marks have specific biological definitions, such as those that mark ‘promoters,’ ‘enhancers’ and the ‘gene body’ in the human genome [3]. The identification of such marks throughout the genome is a common approach to annotating the human epigenome [4, 5].

The application of statistical models to epigenomic datasets consisting of histone modifications, histone variants or chromatin accessibility data has led to the identification of combinatorial histone marks known as chromatin states (CS) [6, 7]. Since conventional algorithms generate every theoretically possible combination, not all of these computed histone mark combinations are found in the human genome; only a fraction of these combinations exists. Initial studies [6] have classified these CSs into five major categories: (1) active intergenic states, (2) large-scale repressed states, (3) promoter-associated states, (4) repetitive states and (5) transcription-associated states [8].

These CSs help functionally annotate the noncoding genome with higher precision without prior knowledge of the genomic elements present. Annotation of the whole genome including noncoding regions using functional biological and genomic labels is an essential step in identifying the regulatory elements of the genome. It allows us to subsequently explore changes in their function under varied cellular conditions such as dysplasia or neoplasia. To this end, several methods have been developed to date that perform CS identification and compare these chromatin labels across the genome between multiple samples or conditions.

## Interrogating chromatin to map chromatin states

A commonly used assay to study chromatin is based on the immunoprecipitation of chromatin using antibodies against proteins bound to it at regions such as histone marks, followed by sequencing (ChIP-seq). More recent methods with fewer limitations that aim to interrogate chromatin are cleavage under targets and release using nuclease (CUT&RUN) and cleavage under targets and tagmentation (CUT&Tag). These methods are considered alternatives to the traditional ChIP-seq methodology [9–11]. To generate a functional annotation map of the human genome using these assays, several different histone modifications should be profiled. Historically, such annotations have been performed by consortia, including the Encyclopedia of DNA Elements (ENCODE), Roadmap Epigenomics, BLUEPRINT, Canadian Epigenetics, Environment and Health Research Consortium (CEEHRC), and International Human Epigenome Consortium (IHEC), as well as by many individual labs. These consortia and labs have systematically mapped regions of transcription, transcription factor association, chromatin structure and histone modification using various assays. These data cover ~80% of the genome [5, 12–17]. Many histone modifications have been examined and are included in these databases. However, several histone modifications have been designated core histone marks (including histone acetylation, or methylation) by most of these consortia [17]. Increasing the number of histone marks assayed can enhance genome annotations and improve the labeling of CSs. However, by including more factors, the number of potential CS labels would increase, which will add to the complexity of the biological interpretation of the generated maps.

Once genome-wide profiles of essential histone proteins are obtained, chromatin marks can be investigated individually, or, using various computational methods, combinatorial histone marks or CSs can be identified. A widely known tool for annotating the noncoding genome is ChromHMM, which identifies CSs throughout the genome [18]. ChromHMM combines multiple epigenomic maps and uses combinatorial and spatial mark patterns to infer the complete annotation for each cell type. Using a multivariate hidden Markov model (HMM) that models the combinatorial presence or absence of each histone modification, ChromHMM learns CSs. These CS signatures are used to generate annotations for each cell type by calculating the most probable CS for each genomic segment. Enrichment analysis of these annotations could enhance biological interpretations of each CS [8]. Other well-known methods that can be utilized to annotate the genome are Segway [19, 20] (a tool that uses a dynamic Bayesian network model to analyze the genome), TreeHMM and hiHMM [20–22]. Any of these methods or other similar methods can be used to identify CSs and annotate the epigenome [23–26].

## Common framework to compare chromatin state maps across epigenomes

Annotation of the genome and mapping of CSs is a significant step toward a better understanding of the functions of genomic segments [27]. The next step is to use this information to investigate changes in essential CSs in normal cells and identify CS changes that perturb cell homeostasis, leading to other cellular conditions such as dysplasia or neoplasia. In this section, we review recent methods that identify CS changes across multiple cell conditions.

To date, several tools have been developed to identify differential CSs under different cellular conditions. These tools include but are not limited to ChromstaR [28, 29], Chromswitch [30], ChromDet [31], ChromDiff [32], SCIDDO [33], differential principal component analysis (dPCA) [34], Epigenome Alignment (EpiAlign) [35] and EpiCompare [36]. Because of the different methodologies of these tools and their varied approaches to calculating differential CSs, their application for different biological conditions varies.

Current methods could be divided in two main groups, (a) methods that can compare CSs maps at the genome-wide level, and (b) methods that can be used for the comparison of CS at a specific genomic region. This classification can define the scope of applicability of each method. In addition, with a few exceptions, almost all methods assume CSs as binary entities as opposed to quantitative variables. Another aspect that determines application of each method is their input formats. CS maps are the usual input of these methods; however, some methods can take singular histone mark as input.

Hereinbelow, we provide a brief, simplified and step-by-step overview of the methodology of each tool along with a general comparison to help readers choose the best and most efficient tool for application to their epigenomic datasets.

### ChromstaR

ChromstaR is an algorithm that computes combinatorial CS changes across several conditions. It uses a multivariate HMM to determine distinct combinatorial histone marks from ChIP-seq experiments. Based on the presence or absence of modifications in each condition, CSs are assigned to all regions across the genome. Aligned reads from each sample are used as input for ChromstaR to carry out the following steps. The algorithm partitions the genome into nonoverlapping bins and calculates read counts that map into each bin. The read count distribution is considered a two-component mixture of zero-inflated negative binomials. One component is the low read count bins, which account for the background noise, and the high read count bins, which represent the signal. A univariate HMM with two hidden states is applied to annotate each bin for the presence or absence of histone marks. To perform joint analysis on all samples simultaneously, a multivariate HMM is used to assign each bin to one of the multivariate components.

ChromstaR has four different modes. (1) The full mode: recommended when the number of marks multiplied by the number of conditions is less than or equal to eight, resulting in a maximum of 256 combinatorial states. (2) The differential mode: outputs significant differences between conditions. The accuracy of the states is lower than that of the full or combinatorial modes. (3) The combinatorial mode: analyzes all measured histone modifications at once. This yields CSs with higher accuracy, and it can be applied for any number of marks and conditions. This mode has a higher false positive rate for differences between conditions compared with the differential or full modes. (4) The separate mode: wherein replicates are processed in a multivariate manner.

In ChromstaR, several theoretically possible combinatorial histone modifications are nearly absent at the genome-wide level. This could be a result of biochemical barriers to the coexistence of two or more histone modifications on the same or adjacent amino acids. Furthermore, some genomic regions are devoid of any measured histone marks, and these are called ‘empty states.’ These regions are subject to change based on the number of histone marks and cell type analyzed.

**Figure 1:**
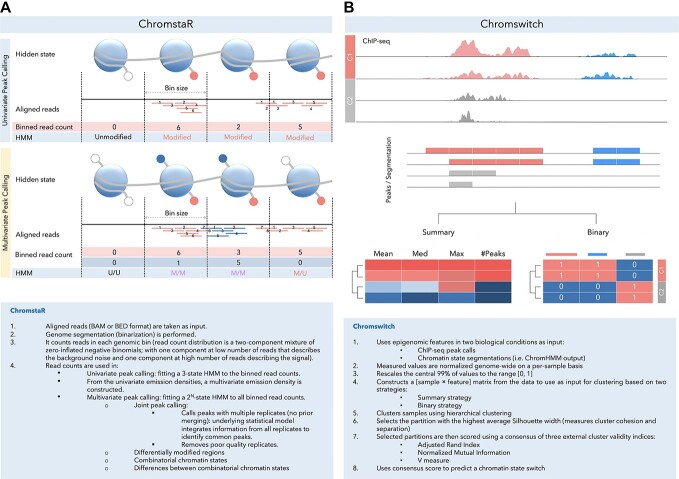
Schematic overview and stepwise algorithm of (**A**) ChromstaR, which is an algorithm that computes combinatorial CS changes across conditions using univariate and multivariate peak calling. Unmodified (U), modified (M) histone protein and (**B**) Chromswitch, which detects tissue-specific CS changes in a defined genomic region using previously generated peaks or CS calls.

Some of the advantages of ChromstaR over other approaches are as follows: (1) it is independent of chromatin segmentation and annotation tools in identifying CSs; (2) the number of CSs does not need to be defined; (3) unlike probabilistic models, each state cannot include multiple and overlapping combinatorial states but is based on patterns of histone modifications; (4) narrow and broad peaks can be analyzed in a combined manner; and (5) replicates can be included as separate experiments with no prior merging. The workflow of ChromstaR is briefly illustrated in Figure 1A, and a more detailed description of the method is shown in the corresponding box.

### Chromswitch

Chromswitch detects spatial, temporal or tissue-specific CS changes in a defined genomic region using peaks or CS calls derived from a DNA–protein or chromatin accessibility profiling methods such as ChIP-seq or DNase-seq. This method takes genomic coordinates along with their corresponding fold changes and *P* values as input. It also provides an option to filter features based on defined thresholds of these values. To adjust for the technical variation between samples, which is common among DNA–protein profiling techniques (i.e. ChIP-seq, CUT&RUN and CUT&Tag), the algorithm normalizes these values per sample at the genome-wide level. Chromswitch then creates a sample-by-feature matrix and performs hierarchical clustering using two different strategies, as shown in Figure 1B. In the first strategy, a statistical summary of all peaks present in the genomic region is stored in the feature vector for that sample (summary strategy). In the second strategy, the presence or absence of each unique peak in a sample is stored in a binary vector (binary strategy). Once the summary and binary matrices are constructed, Chromswitch then clusters samples using hierarchical clustering and selects a partition of samples with the highest average Silhouette width. Next, to measure the consistency between the predicted sample partition from the previous step and the ground-truth sample partition, the algorithm computes a consensus score defined by the average of three cluster validity indices: the adjusted Rand index (ARI), the normalized mutual information score (NMI) and the V measure. The tool then uses this score to rank CS changes.

An advantage of Chromswitch is its applicability to small sample sizes and high class imbalance. The algorithm also performs well for histone marks with sparse signals and broad domains.

### ChromDet

ChromDet is a method to classify samples from multiple cell types by leveraging unique epigenomic features that are common across subsets of samples. Given the hypothesis that cellular phenotypes are directly related to their epigenetic composition and the contribution of chromatin changes during differentiation to cell fate, ChromDet aims to identify chromatin changes across the genome among various cell types. The algorithm can identify chromatin drivers corresponding to a set of regions that are altered during phenotypic transitions. ChromDet is a modified version of an earlier tool, S3det [37]. It is based on multiple correspondence analysis (MCA), which generates the chromatin sample space, defined as the space formed by a set of highly informative components resulting from the MCA on the vectors of the CSs in the samples. MCA is essentially equivalent to PCA when processing qualitative data rather than continuous variables. Unsupervised *k*-means clustering is then performed on the chromatin sample space for a range of groups (*n* = 2–50), and the optimal number of clusters is determined using the Calinski–Harabasz Index (CHI). A second space, the chromatin region space, is also generated using MCA analysis and is a projection of the vectors reflecting every CS combination into the MCA space. Chromatin determinant regions (CDRs) are obtained by choosing those genomic regions that overlap with cluster sample fingerprints. The cluster sample fingerprints are vectors representing chromatin patterns that are unique to each group of samples identified by chromatin sample space. Each of the epigenomic regions is then associated with its nearby fingerprint in the chromatin region space. Lastly, CDRs are defined as genomic positions for which all CSs are among the top 10 ranked shortest distances to their fingerprints in the chromatin region space. This combination of fingerprints partitions the sample clusters.

ChromDet can be utilized to detect CS changes for DNA–protein profiling methods such as ChIP-seq, CUT&RUN and CUT&Tag. Similar to other approaches, for these datasets, the raw data should be processed using a common pipeline (i.e. the ENCODE ChIP-seq pipeline or CUT&RUNTools), and peaks and other output files are generated [38]. Next, the processed data undergoes segmentation and genome annotation. The output can then be used by ChromDet to detect sample clusters, allowing the identification of those regions that are important for establishing segregation of the samples and identification of CDRs, which will define inter-cluster epigenomic changes.

**Figure 2:**
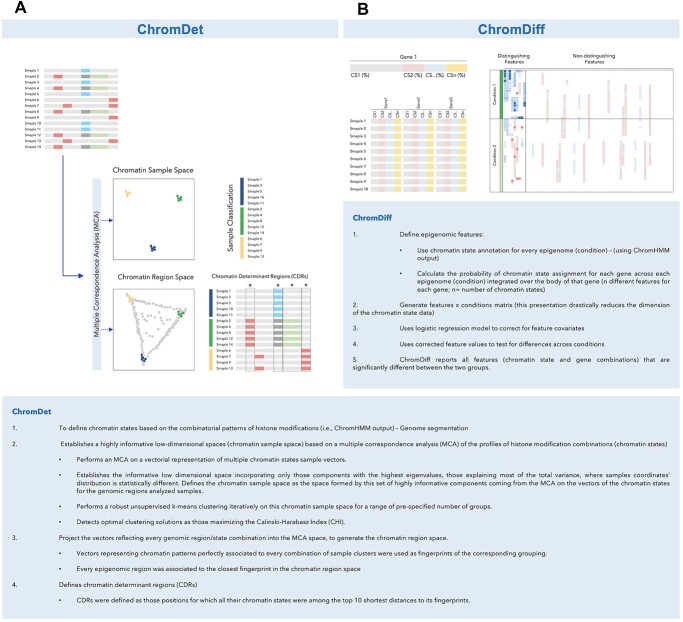
Schematic overview and stepwise algorithm of (**A**) ChromDet, which classifies multiple cell types by subtype-specific epigenomic features, and (**B**) ChromDiff, a statistical algorithm for groupwise CS comparison.

One of the additional features of ChromDet is defining an epigenomic divergence index by calculating the ratio of CDRs with a CS different from the healthy cell condition. This feature represents the level of divergence of a sample from the healthy sample space, which is based on normal samples. The ChromDet workflow is illustrated in Figure 2A.

### ChromDiff

ChromDiff is a statistical pipeline for groupwise CS comparisons. It is broadly applicable for epigenomic comparisons and for studying CS differences at the genome-wide level. ChromDiff addresses the question of how CS segmentation differs under various conditions or tissue types. It captures discriminating epigenomic features between two groups by comparing their CS patterns. The algorithm generates an information-theoretic representation of the epigenomic data and applies a covariate correction for large-scale sample analysis. ChromDiff calculates statistics of recurrent CS changes and associates the identified regions with their corresponding protein-coding genes. The resulting gene sets can be further utilized to explore dysregulation in biological pathways between the two groups. Because ChromDiff focuses on gene body regions, downstream analyses can be performed on the resulting gene sets, such as gene set enrichment analysis to investigate biological processes associated with epigenomic differences. The expression level of differential genes can also be compared with the epigenomic features. Moreover, hierarchical clustering can be applied to identify cluster-specific gene sets.

This method takes a gene-centric approach and defines epigenomic features by calculating the probability of CS labels for each gene in each sample. This representation significantly reduces the CS data and at the same time preserves the information necessary to calculate the information theory metrics. Next, the algorithm generates a feature × sample matrix and applies a logistic regression model to correct for feature covariates. ChromDiff then uses the corrected feature values to detect differences between the two groups using a nonparametric Mann–Whitney–Wilcoxon test, Student’s *t* test or *F* test with multiple hypothesis correction. Finally, the pipeline outputs significantly different CSs and gene combinations between the groups. ChromDiff can also be used to cluster differential regions with similar CS signatures into distinct subgroups, thereby highlighting subgroup-specific gene set enrichment and expression patterns.

An advantage of ChromDiff is its ability to correct for external covariate factors, which is an essential step in differential analysis when using datasets with experimental and sample differences as well as variation due to batch effects. This feature was not part of prior algorithms such as dPCA. The ChromDiff workflow is briefly illustrated in Figure 2B.

### Sciddo

**Figure 3:**
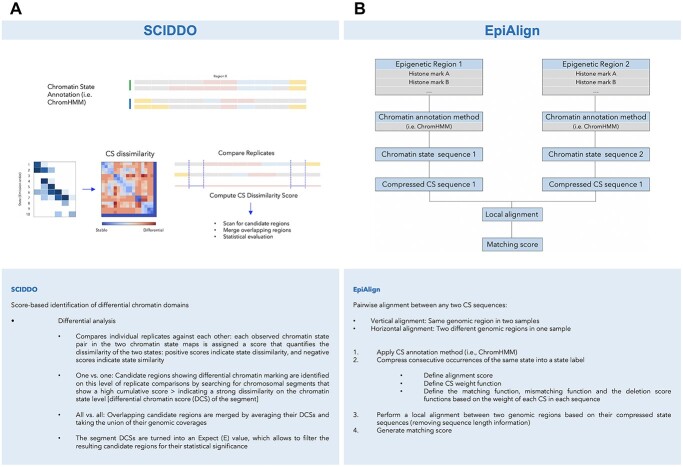
Schematic overview and stepwise algorithm of (**A**) SCIDDO, a score-based method for the identification of DCD by quantifying the dissimilarity of the two states, and (**B**) EpiAlign, a pairwise alignment-based tool that compares CS sequences from several datasets and identifies locally aligned chromatin regions.

SCIDDO is a score-based method that identifies differential chromatin domains (DCD). It uses CS segmentation maps to compute CS dissimilarities in multiple groups or across various cell types. SCIDDO essentially has two main steps. In the first step, CS emission probabilities are used to calculate pairwise CS dissimilarities. The second step is a differential analysis by comparing individual replicates from one group with the replicates from a second group or biological condition. Next, at each position of the genome, if a replicate from group *x* is in state *i* and a replicate from group *y* is in state *j*, that genomic position is assigned to a precalculated score *s^ij^* that measures the dissimilarity between states *i* and *j*. Positive scores indicate dissimilarity and negative scores indicate similarity between states. In this way, chromosomal segments with high cumulative scores are identified. The cumulative score is referred to as the differential chromatin score (DCS), which is an indicator of the dissimilarity between CSs. Overlapping identified regions are summarized across all samples by the average of their DCS and by applying the union of their coverage. DCSs for each segment are converted into expected values (*E*) to allow the ranking of candidate regions based on their statistical significance and additional analysis. Furthermore, the identified DCDs correlate with differentially expressed genes between groups. One advantage of SCIDDO over similar methods is its applicability on a genome-wide scale and its high performance in a limited number of samples. The workflow of SCIDDO is illustrated in Figure 3A, and a detailed description of the method is shown in the corresponding box.

### EpiAlign

EpiAlign is a pairwise alignment-based tool that compares CS sequences learned from several datasets and identifies locally aligned chromatin regions. The algorithm compares pairs of CS sequences by computing a local alignment score. The program detects the alignment between CS sequences using both mismatch and deletion scores. These scores are based on the weight of each CS in the sequence. A CS segmentation method is first applied to the datasets to obtain a single-track CS sequence. Consecutive occurrences of a similar CS on each epigenome are compressed into one individual state label, and then EpiAlign performs a local alignment-based on the compressed state labels of the two genomic regions. Compressed state sequences are generated since most uncompressed sequences contain long repetitive CSs that are mostly quiescent or low. Therefore, this method allows one to focus on the CS pattern rather than a single CS that spans a long genomic region. To perform CS sequence alignment, EpiAlign uses a modified Smith–Waterman algorithm, which is based on triplets of matches, mismatches, and gaps. EpiAlign can extract similar CS pattern from one epigenome or across multiple epigenomes. EpiAlign uses a vertical alignment strategy to compare CS sequences of coding genes across multiple epigenomes. The vertical alignment strategy is applied to the compressed CS labels assigned to the genomic region of interest. This approach compares alignment scores across multiple epigenomes.

A notable feature of EpiAlign compared with that of other similar methods is its effective use of sequential information of CSs by generating compressed CS labels. Sequential CSs harbor essential information on the mechanisms of gene regulation [18]. EpiAlign is briefly described in Figure 3B.

### dPCA

dPCA is an algorithm that uses a small number of principal components to infer differential protein–DNA interactions from multiple ChIP-seq datasets from two biological conditions. dPCA uses unsupervised pattern discovery to identify the main patterns of differences between the conditions using differential principal components (dPCs) that represent the covariation pattern of quantitative signals among several proteins or histone marks. The algorithm then identifies differential genomic loci for each of the main dPCs and ranks them based on the magnitude of differences. Statistical significance for each locus is determined by the comparison of differences between conditions with the background variation among replicates.

**Figure 4:**
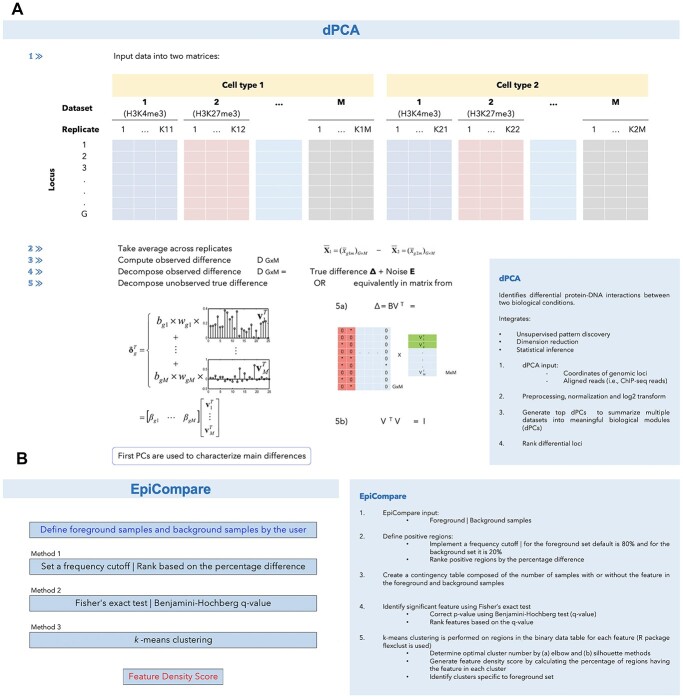
Schematic overview and stepwise algorithm of (**A**) dPCA, an algorithm that uses principal components to infer differential protein–DNA interactions from ChIP-seq datasets from two biological conditions. *M* is the number of datasets for each condition (i.e. cell type); *K* is the number of replicates; *G* is the number of loci; *D* is the matrix with rows corresponding to loci and columns to datasets; *B* is the matrix of group coefficients *β*_gj_; *V* is a matrix representing a covariation pattern of binding intensities among multiple samples, and (**B**) EpiCompare, an approach that identifies genome segments with unique epigenomic features across different cell types.

dPCA is a deterministic algorithm that does not require random initialization or the ad hoc choice of parameters, which some other methods such as *k*-means clustering use. This characteristic makes the patterns discovered by dPCA more reproducible. The workflow of dPCA is briefly illustrated in Figure 4A with a concise description of the method in the corresponding box.

### EpiCompare

EpiCompare is composed of three algorithms that identify genome segments with unique epigenomic features across different cell types. For these algorithms, users must define foreground samples, or the group for which unique epigenomic features are being sought, and background samples, or the group of samples against which the foreground samples are being compared. EpiCompare identifies chromatin regions enriched in the foreground group compared with the background samples.

The first algorithm sets a frequency cutoff for each feature, and samples having the feature in either the foreground or background set are calculated (the default cutoff is 80% in the foreground samples and 20% in the background samples). Features that fulfill these criteria are considered positive regions and are ranked based on the percentage difference. The second algorithm applies Fisher’s exact test between the number of samples with the specific feature in the foreground and the background samples. All identified features are ranked based on the calculated Benjamini–Hochberg *q* values. The third algorithm performs *k*-means clustering based on the Jaccard index distance on regions in the binary data table for each feature. The optimal number of clusters is determined by the elbow and Silhouette methods. Feature density is calculated for each cluster, and it is defined as the percentage of regions with the feature in each cluster. The clusters unique to each cell type are defined as those with a higher feature density compared with that of the background set.

EpiCompare allows the identification of group-specific epigenomic features such as different types of tissue or lineage-specific regulatory elements. The algorithm allows users to compare different combinations of epigenomic datasets either in their data or in Roadmap Epigenomics. Furthermore, EpiCompare output can be readily visualized in the WashU Epigenome Browser. The EpiCompare workflow is briefly illustrated in Figure 4B, and a more detailed description of the method is shown in the corresponding box.

## Discussion and future perspectives

In this review, we discussed various methods that identify the epigenomic features that are unique to a group of samples. Despite differences in methodologies, these algorithms detect differential chromatin patterns in multiple groups of samples. Finding such patterns in biological or pathological cell state transitions can aid the understanding of the process of cell transformation. For instance, such analysis could be informative about the processes of cellular differentiation, the impact of environmental stimuli, or even tumorigenesis. The transition of normal cells to cancer cells is accompanied by crucial changes in CSs in which various transcriptionally active regions are suppressed or vice versa [39–42]. Tumorigenesis may involve cells gaining active CSs in previously empty or quiescent chromatin regions, or, conversely, regions with active CSs may lose histone marks.

**Table 1:** Comparison of differential chromatin analysis methods

Method	Input	Type of input		Generates (feature × sample) matrix	Chromatin state variable (input)	User platform|language	Scope of application
		Singular histone mark	Chromatin state				
Chromswitch	Peak calls or CS generated by other tools	✔	✔	✔	Binary|scale	R package	Genomic region
ChromDet	CS generated by other tools		✔	Generates chromatin sample|chromatin region space	Binary	Command line	Genome-wide
ChromDiff	CS generated by other tools		✔	✔	Binary	R|bash	Genomic regions (coding genes)
SCIDDO	CS generated by other tools		✔	N/A	Quantitative scale	Python	Genome-wide
EpiAlign	CS generated by other tools		✔	N/A	Binary	Web tool	Genomic region
EpiCompare	CS generated by other tools		✔	✔	Binary	R package|web tool	Genome-wide
ChromstaR	Aligned reads	✔		N/A	Binary	R package	Genome-wide
dPCA	Aligned reads	✔		N/A	Binary	Command line	Genomic region

*Notes*: Parameters such as the input (peak calls versus CS segmentation generated from other tools), the type of input that can be used (singular histone mark versus chromatin state), the generation of feature × sample matrix in the method, binary versus quantitative analysis of chromatin states, the programming language or user platform, and the scope of the application of each tool are compared.

**Figure 5:**
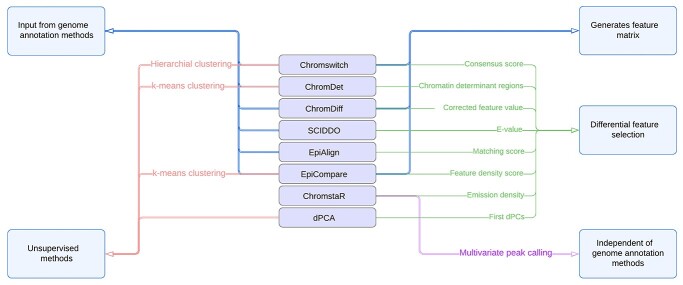
Prominent features of differential chromatin analysis methods. The unique differential feature selection algorithms and clustering methods are indicated. The clustering methods are depicted in red. ChromDet and EpiCompare use *k*-means clustering, whereas Chromswitch uses hierarchical clustering. Algorithms that are dependent on a genome annotation method are marked in blue, on the left side. ChromstaR is the only method that performs multivariate peak calling and is independent of genome annotation methods. Algorithms that generate feature matrices are marked in blue on the right side of the diagram. Differential feature selection assessment scores (method) are indicated in green for each algorithm.

Although most of the described methods use epigenomic features that have previously been inferred from each sample as input; however, ChromstaR and dPCA can take aligned reads and process them further to perform differential analysis (Table 1). The input data in many cases are peak files from a DNA–protein profiling or chromatin accessibility assay (e.g. ChIP-seq, CUT&RUN and CUT&Tag) generated by a peak calling tool or CS segmentation files generated by a genome annotation tool (e.g. ChromHMM). The only tool reviewed here that performs joint peak calling is ChromstaR, which uses a multivariate HMM to determine the number of CSs, whereas, in tools such as ChromHMM, the user must determine the number of CSs. However, mathematical models such as the Akaike Information Criterion (AIC) and the Bayes Information Criterion (BIC) automatically determine a particular CS number and perform this step instead of leaving it to the user. Three of these methods generate an epigenomic [feature × sample] matrix (Chromswitch, ChromDiff and EpiCompare), whereas ChromDet utilizes MCA, a method similar to PCA but for categorical data, to generate chromatin sample space that is used with the chromatin region space to define group-specific patterns. Conversely, SCIDDO calculates a dissimilarity CS score. Whereas other methods consider CS similarity as a binary value, SCIDDO applies a quantitative (score-based) scale to determine CS differences. The SCIDDO algorithm identifies DCD that correlates well with differentially expressed genes. Another approach used to identify differential CSs is calculating a local alignment score to compare multiple epigenomes. EpiAlign uses single-track CS sequences and compresses them, allowing a focus more on CS pattern discovery by removing the impact of CS length, which can obscure such patterns in long repetitive regions of the genome [35]. A compressed CS also increases the model performance and improves the alignment.

Some of these methods only perform region-based analysis. Chromswitch, EpiAlign, ChromDiff and dPCA can be applied to a user-specified genomic region, whereas other methods apply a genome-wide approach. Although ChromDiff can be applied to any region, the focus of the algorithm is on gene bodies to improve the interpretability of the results. Furthermore, using standard statistical tests, such as the nonparametric Mann–Whitney–Wilcoxon test as in ChromDiff for the identification of differential CSs, restrains the algorithm to reach a sufficient statistical power only by including a large number of replicates in each group. This also applies to algorithms used in ChromDet and dPCA, making these methods more suitable for larger numbers of samples, while it can also be a limitation of these methods to be used for epigenomic datasets with fewer number of samples.

Figure 5 and Table 1 summarize the prominent features of differential CS methods along with some of the commonalities between the different methods. The unique features of each method can facilitate the choice of the appropriate tool for the analysis of specific epigenomic datasets.

None of the tools discussed in this review have been benchmarked for application in single-cell epigenomic datasets. The sparse nature of single-cell epigenomic datasets requires the development of new models and algorithms tuned for such datasets. The ChromVAR and PRISM algorithms are examples of methods specifically developed for single-cell assay for transposase-accessible chromatin (scATAC-seq) datasets, which could be adapted for CS comparisons at the single-cell level [43–45].

**Key Points**

- Combinatorial histone marks on the noncoding genome can identify chromatin states (CS).
- Identification of CSs across the genome enables functional annotation of the epigenome.
- Various computational methods can be used to interrogate CS changes among different cellular conditions.
- Stepwise algorithms and distinct features of each method can lead to a better understanding and applicability of these tools for various epigenomic datasets.

## Supplementary Material

Summary_Box_R3_bbac439

## Author Biographies

**Elias Orouji** Staff Scientist and Epigenomics Lead at the Princess Margaret Cancer Centre, University Health Network in Toronto, Canada.

**Ayush T Raman** Postdoctoral Fellow at the Broad Institute of MIT and Harvard.
