# Supplementary material for: Computational methods to explore chromatin state dynamics
Source: Brief Bioinform. 2022 Oct 7;23(6):bbac439. doi: 10.1093/bib/bbac439 (PMC9677473; doi:10.1093/bib/bbac439)
Supplement: Summary_Box_R3_bbac439 [file summary_box_r3_bbac439.docx]

**Supplementary information – Summary box for each method**

**ChromstaR**

1. Aligned reads (BAM or BED format) are taken as input.
2. Genome segmentation (binarization) is performed.
3. It counts reads in each genomic bin (read count distribution is a two-component mixture of zero-inflated negative binomials; with one component at low number of reads that describes the background noise and one component at high number of reads describing the signal).
4. Read counts are used in:
   - Univariate peak calling: fitting a 3-state HMM to the binned read counts.
   - From the univariate emission densities, a multivariate emission density is constructed.
   - Multivariate peak calling: fitting a 2^N^-state HMM to all binned read counts.
     - Joint peak calling:
       - Calls peaks with multiple replicates (no prior merging): underlying statistical model integrates information from all replicates to identify common peaks.
       - Removes poor quality replicates.
     - Differentially modified regions
     - Combinatorial chromatin states
     - Differences between combinatorial chromatin states

**Chromswitch**

1. Uses epigenomic features in two biological conditions as input:
   - ChIP-seq peak calls
   - Chromatin state segmentations (i.e., ChromHMM output)
2. Measured values are normalized genome-wide on a per-sample basis
3. Rescales the central 99% of values to the range [0, 1]
4. Constructs a [sample × feature] matrix from the data to use as input for clustering based on two strategies:
   - Summary strategy
   - Binary strategy
5. Clusters samples using hierarchical clustering
6. Selects the partition with the highest average Silhouette width (measures cluster cohesion and separation)
7. Selected partitions are then scored using a consensus of three external cluster validity indices:
   - Adjusted Rand Index
   - Normalized Mutual Information
   - V measure
8. Uses consensus score to predict a chromatin state switch

**ChromDet**

1. To define chromatin states based on the combinatorial patterns of histone modifications (i.e., ChromHMM output) – Genome segmentation

2. Establishes a highly informative low-dimensional spaces (chromatin sample space) based on a multiple correspondence analysis (MCA) of the profiles of histone modification combinations (chromatin states)

- - Performs an MCA on a vectorial representation of multiple chromatin states sample vectors.
  - Establishes the informative low dimensional space incorporating only those components with the highest eigenvalues, those explaining most of the total variance, where samples coordinates’ distribution is statistically different. Defines the chromatin sample space as the space formed by this set of highly informative components coming from the MCA on the vectors of the chromatin states for the genomic regions analyzed samples.
  - Performs a robust unsupervised k-means clustering iteratively on this chromatin sample space for a range of pre-specified number of groups.
  - Detects optimal clustering solutions as those maximizing the Calinski-Harabasz Index (CHI).

3. Project the vectors reflecting every genomic region/state combination into the MCA space, to generate the chromatin region space.

- - Vectors representing chromatin patterns perfectly associated to every combination of sample clusters were used as fingerprints of the corresponding grouping.
  - Every epigenomic region was associated to the closest fingerprint in the chromatin region space

4. Defines chromatin determinant regions (CDRs)

- - CDRs were defined as those positions for which all their chromatin states were among the top 10 shortest distances to its fingerprints.

**ChromDiff**

1. Define epigenomic features:

- - Use chromatin state annotation for every epigenome (condition) – (using ChromHMM output)
  - Calculate the probability of chromatin state assignment for each gene across each epigenome (condition) integrated over the body of that gene (n different features for each gene; n= number of chromatin states)

2. Generate features x conditions matrix (this presentation drastically reduces the dimension of the chromatin state data)

3. Uses logistic regression model to correct for feature covariates

4. Uses corrected feature values to test for differences across conditions

5. ChromDiff reports all features (chromatin state and gene combinations) that are significantly different between the two groups.

**SCIDDO**

Score-based identification of differential chromatin domains

• Differential analysis

- - Compares individual replicates against each other: each observed chromatin state pair in the two chromatin state maps is assigned a score that quantifies the dissimilarity of the two states: positive scores indicate state dissimilarity, and negative scores indicate state similarity
  - One vs. one: Candidate regions showing differential chromatin marking are identified on this level of replicate comparisons by searching for chromosomal segments that show a high cumulative score > indicating a strong dissimilarity on the chromatin state level [differential chromatin score (DCS) of the segment]
  - All vs. all: Overlapping candidate regions are merged by averaging their DCSs and taking the union of their genomic coverages
  - The segment DCSs are turned into an Expect (E) value, which allows to filter the resulting candidate regions for their statistical significance

**EpiAlign**

Pairwise alignment between any two CS sequences:

- Vertical alignment: Same genomic region in two samples
- Horizontal alignment: Two different genomic regions in one sample

1. Apply CS annotation method (i.e., ChromHMM)
2. Compress consecutive occurrences of the same state into a state label
   - Define alignment score
   - Define CS weight function
   - Define the matching function, mismatching function and the deletion score functions based on the weight of each CS in each sequence
3. Perform a local alignment between two genomic regions based on their compressed state sequences (removing sequence length information)
4. Generate matching score

**dPCA**

Identifies differential protein-DNA interactions between two biological conditions.

Integrates:

- Unsupervised pattern discovery
- Dimension reduction
- Statistical inference

1. dPCA input:
   - Coordinates of genomic loci
   - Aligned reads (i.e., ChIP-seq reads)
2. Preprocessing, normalization and log2 transform
3. Generate top dPCs to summarize multiple datasets into meaningful biological modules (dPCs)
4. Rank differential loci

**EpiCompare**

1. EpiCompare input:
   - Foreground | Background samples
2. Define positive regions:
   - Implement a frequency cutoff | for the foreground set default is 80% and for the background set it is 20%
   - Ranke positive regions by the percentage difference
3. Create a contingency table composed of the number of samples with or without the feature in the foreground and background samples
4. Identify significant feature using Fisher’s exact test
   - Correct p-value using Benjamini-Hochberg test (q-value)
   - Rank features based on the q-value
5. k-means clustering is performed on regions in the binary data table for each feature (R package flexclust is used)
   - Determine optimal cluster number by (a) elbow and (b) silhouette methods
   - Generate feature density score by calculating the percentage of regions having the feature in each cluster
   - Identify clusters specific to foreground set
